# The Impact of Sedative Choice in the Management of Aneurysmal Subarachnoid Hemorrhage: A Scoping Review

**DOI:** 10.1007/s12028-024-02111-1

**Published:** 2024-09-12

**Authors:** James Then, Samuel Tawfik, Timothy Law, Alastair Brown, Vanessa Carnegie, Andrew Udy, Toby Jeffcote

**Affiliations:** 1 Department of Intensive Care, The Royal Melbourne Hospital, Melbourne, VIC Australia; 2 The Victorian Brain and Spine Centre, St. Vincent’s Hospital, Melbourne, VIC Australia; 3 Department of Intensive Care, St. Vincent’s Hospital, Melbourne, VIC Australia; 4 Australia and New Zealand Intensive Care Research Centre, Monash University, Melbourne, Australia; 5 Department of Critical Care, University of Melbourne, Melbourne, Australia; 6 Department of Intensive Care, Sir Charles Gairdner Hospital, Nedlands, WA Australia; 7 Department of Intensive Care, The Alfred Hospital, Melbourne, VIC Australia

**Keywords:** Subarachnoid hemorrhage, Ruptured intracranial aneurysm, Deep sedation, Conscious sedation, Opioid, Opiate, Morphine, Ketamine, Fentanyl, Dexmedetomidine, Clonidine, Adrenergic alpha agonist, Medetomidine, Benzodiazepines, Diazepam, Midazolam, Barbituarates, Thiopental, Phenobarbital

## Abstract

Aneurysmal subarachnoid hemorrhage (aSAH) is characterized by high mortality and morbidity. This scoping review assesses the current evidence regarding the use of sedatives and analgesics in the acute intensive care unit management of aSAH. We conducted a systematic search of Ovid MEDLINE, Ovid Embase, Ovid EmCare, APA PsycInfo, CINAHL, and the Cochrane Database of Systematic Reviews from inception to June 2023. Studies were included if they enrolled intensive care unit patients aged 18 or older with a significant proportion (> 20%) who had aSAH and evaluated the impact of one or more commonly used analgosedatives on physiological parameters in the management of aSAH. The methodological quality of the studies was assessed using the Methodological Index for Nonrandomized Studies score. Of 2,583 articles, 11 met the inclusion criteria. The median sample size was 47 (interquartile range 10–127), and the median Methodological Index for Nonrandomized Studies score was 9.5 (interquartile range 8–11). The studies’ publication years ranged from 1980 to 2023. Dexmedetomidine and ketamine showed potential benefits in reducing the incidence of cortical spreading depolarization and delayed cerebral ischemia. Propofol and opioids appeared safe but lacked robust evidence for efficacy. Benzodiazepines were associated with increased delayed cerebral ischemia–related cerebral infarctions and cortical spreading depolarization events. The evidence available to guide the use of analgosedative medications in aSAH is critically inadequate. Dexmedetomidine and ketamine warrant further exploration in large-scale prospective studies because of their potential benefits. Improved study designs with consistent definitions and a focus on patient-centered outcomes are necessary to inform clinical practice.

## Introduction

Aneurysmal subarachnoid hemorrhage (aSAH), is characterized by high mortality and morbidity, which are directly related to the severity of disease [1]. Approximately 50% of patients with aSAH die within 30 days of aneurysm rupture, and a significant proportion of survivors suffer from debilitating complications [1]. aSAH can be of varying severity, which is largely driven by the volume of blood released into the subarachnoid space and any delayed pathophysiological manifestations of the disease. Initial management focuses on securing the aneurysm and prevention of rebleeding. Subsequent management focuses on management of hydrocephalus, and delayed cerebral ischemia (DCI), which is particularly important with more severe cases.

The prognosis of patients with aSAH is significantly impacted by the occurrence of DCI [2, 3]. Prevention and treatment of DCI remains challenging, despite recent advances in the understanding of the underlying pathophysiological processes [4]. Initial observations of a link between subarachnoid hemorrhage, spasm of the proximal cerebral arteries and DCI led to the conclusion that proximal vasospasm was the sole cause of DCI. However, evidence of ischemic damage remote from perfusion territories of spastic arteries and the failure of endothelin receptor antagonists to improve outcomes from aSAH despite reductions in angiographic vasospasm [5, 6] reinforced the conclusion that DCI can occur even in the absence of spasm of the proximal cerebral arteries. DCI is increasingly recognized to result from a complex combination of factors including, angiographic vasospasm, microvascular constriction, blood brain barrier disruption, formation of microthrombi, inflammation and cortical spreading ischemia [2, 7].

This evolving understanding of the factors underpinning DCI should prompt a renewed focus on the clinical management of patients with severe aSAH and a reassessment of how our management strategies interact with the pathophysiological processes involved [4]. One of the key management modalities for severe aSAH is sedation. It is essential for safe mechanical ventilation in comatose patients but also influences clinical parameters such as arterial blood pressure, cerebral blood flow and cerebral metabolic coupling [8]. Additionally, sedation may contribute to reducing cerebral oxygen consumption [8]. Sedative and analgesic agents also influence N-methyl-D-aspartate receptor–mediated cortical spreading depolarization and thus may offer a degree of neuroprotection [9]. Analgo-sedation is therefore an integral part of neurocritical care management of acute severe aSAH. Additionally, the unique pathophysiology of aSAH means that the clinical goals of aSAH management are distinct from those of other acute brain injuries such as traumatic brain injury, for which other reviews of sedative practice are available [10]. Despite the widespread use of these agents, established protocols do not provide recommendations for this area of practice [11] and to our knowledge the evidence base for sedation strategies in aSAH has not been systematically assessed.

This scoping review aims to present a comprehensive overview of the current evidence regarding the use of sedatives and analgesics in the acute intensive care unit (ICU) management of aSAH. Of note, despite the extensive application of these sedatives, there remains a lack of large-scale prospective trials with sufficient statistical power to discern their impact on key patient-centered outcomes, such as all-cause mortality and longer-term neurological function. As such, our objective was to summarize the known physiological effects of sedation in aSAH and identify potential candidate sedatives for future large-scale clinical trials.

## Objectives

Our objectives were to perform a scoping review of analgosedative agents available for the acute management of aSAH and review three key questions:

1. Is there evidence for the use of specific analgosedative agents in the management of aSAH?
2. What is the strength and methodological quality of this evidence?
3. Do any of the available agents offer potential advantages in terms of safety and physiological effects?

## Methods

Methods for inclusion and analysis of studies were prespecified in a protocol developed in accordance with the most recent Preferred Reporting Items for Systematic Reviews and Meta-analyses (PRISMA) [12] and the Cochrane collaboration guidelines [13]. The protocol was prospectively registered with Open Science Framework on May 26th, 2023.

### Search Strategy

**Fig. 1:**
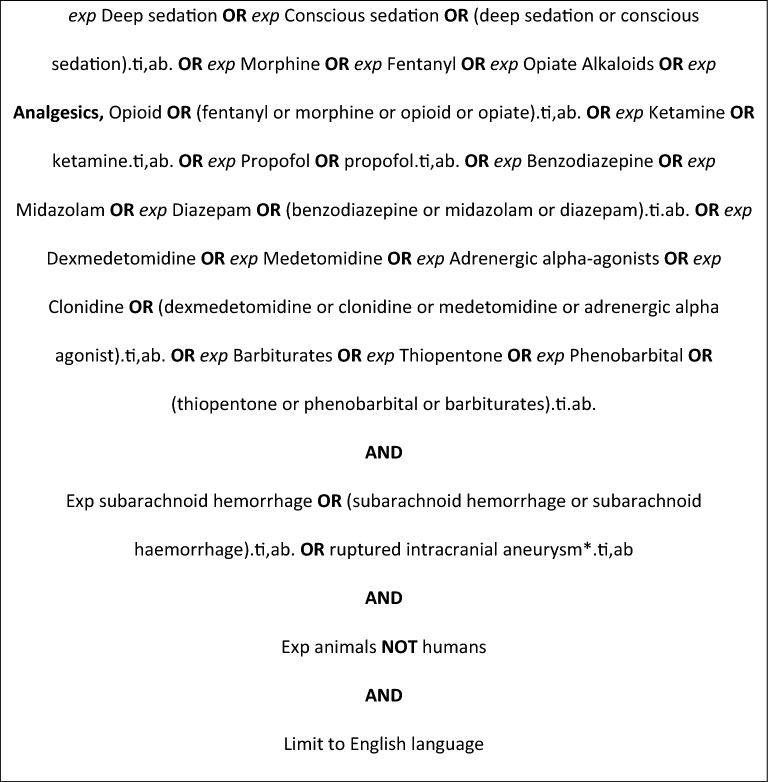
Search strategy

Electronic searches were performed on Ovid MEDLINE, Ovid Embase, Ovid EmCare, APA PsycInfo, CINAHL, and the Cochrane Database of Systematic Reviews from their dates of inception to June 2023. Searches were performed with multiple terms as Medical Subject Headings (MeSH) terms and keywords derived from the key concepts of “sedation” and “subarachnoid hemorrhage” using a combination of keywords and subject headings in Table 1 (Fig. 1) (see the “Appendix”). Citations and abstracts were retrieved. A hand search of the bibliographies was also performed to identify relevant articles missed by the electronic search.

**Table 1:** Key search concepts

	Concept 1	Concept 2
Key Concept	Sedation	Subarachnoid hemorrhage
Subject Heading	Deep sedation	Subarachnoid hemorrhage
	Conscious sedation	Subarachnoid heaemorrhage
		Ruptured intracranial aneurysm
Key Concept	Analgaesia	
Subject Heading	Opioid	
	Opiate	
Key Concept/ Subject Heading	Morphine	
	Ketamine	
	Fentanyl	
Key Concept	Dexmedetomidine	
Subject Heading	Dexmedetomidine	
	Clonidine	
	Adrenergic alpha agonists	
	Medetomidine	
Key Concept/ Subject Heading	Benzodiazepines	
	Diazepam	
	Midazolam	
	Barbiturates	
	Thiopental	
	Phenobarbital

### Study Selection and Appraisal

Articles were included if they enrolled ICU patients, aged 18 years or older, with a significant proportion (> 20%) who had aSAH and evaluated the impact of one or more commonly used analgosedatives on physiological parameters in the management of aSAH. Commonly used analgosedatives were defined as propofol, opioids, benzodiazepines, barbiturates, ketamine, and alpha-2 agonists. Articles were excluded if they investigated anesthetic gases because they are not widely used in the ICU. Eligible studies needed to include at least one outcome related to DCI, including, vasospasm incidence, intracranial pressure (ICP), cerebral perfusion pressure (CPP), cortical spreading depolarization (CSD), 28-day mortality, length of stay in the ICU, length of stay in the hospital, Glasgow Outcome Scale (GOS) score, modified Rankin Scale (mRS) for neurologic disability and discharge destination. Randomized controlled trials and observational studies were included, whereas reviews, case reports, editorials, and conference proceedings were excluded. Articles were also excluded if they were not in English. The articles were reviewed for inclusion or exclusion independently by two authors (JT and ST), and disagreements were resolved by group consensus.

The selected studies were appraised for risk of bias and methodological integrity using the Methodological Index for Nonrandomized Studies (MINORS) score [14], which is applicable to both randomized and nonrandomized interventional studies [15]. The MINORS scores are classified as low (5–8), moderate (9–12), and high (13–16). Studies were included in qualitative summary if they achieved a MINORS score exceeding 7 because this threshold represents a 50% score on the nonrandomized component of the MINORS scale and aligns with benchmarks used in similar literature reviews [16]. Disagreements were resolved by consensus approach.

### Data Analysis

**Fig. 2:**
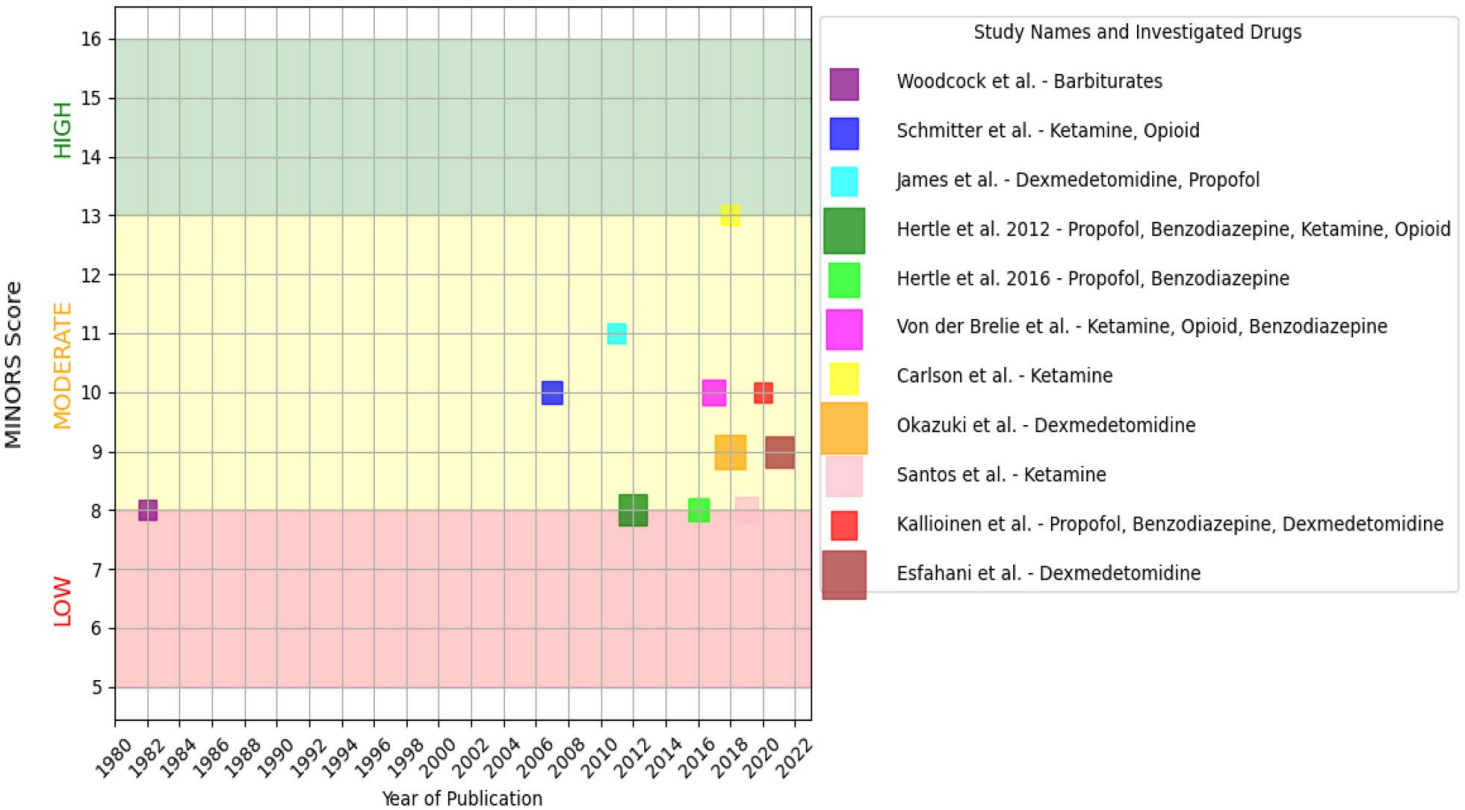
Timeline and quality of publication

Given the diversity in outcome measures and effects across the studies, we decided against a conventional data synthesis or meta-analysis. Instead, to effectively illustrate the current evidence landscape, we categorized the studies based on the class of sedative used and primary outcome of the study. These studies were then graphed to showcase the quality of evidence and publication dates (Fig. 2).

## Results

**Fig. 3:**
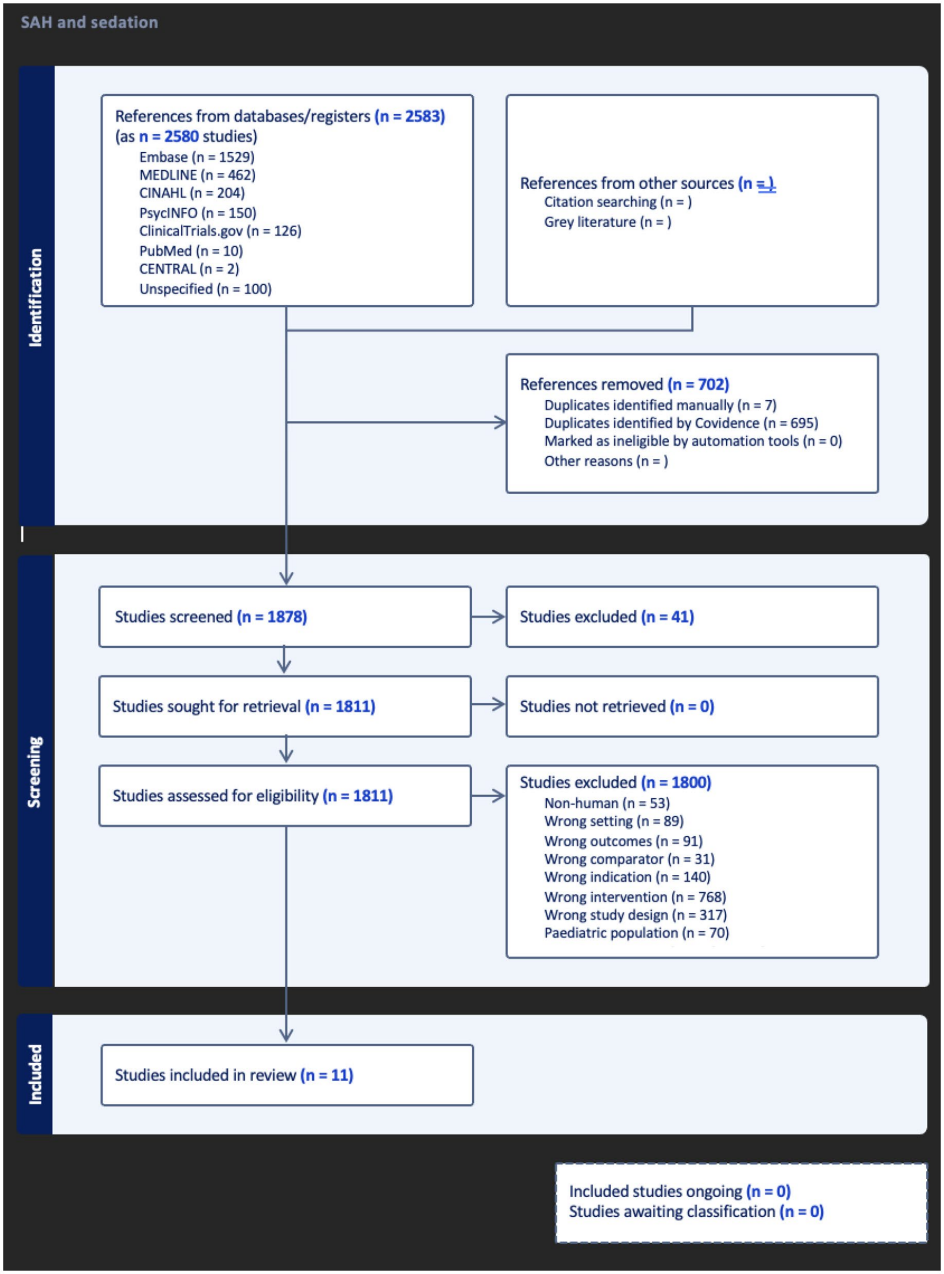
PRISMA flow diagram

A total of 2,583 articles were retrieved from database searches. There were 702 duplicate articles, 41 irrelevant articles, and 1,841 articles that did not meet the inclusion criteria. Eleven articles met the inclusion criteria and were ultimately included in this review. The PRISMA diagram is shown in Fig. 3.

**Fig. 4:**
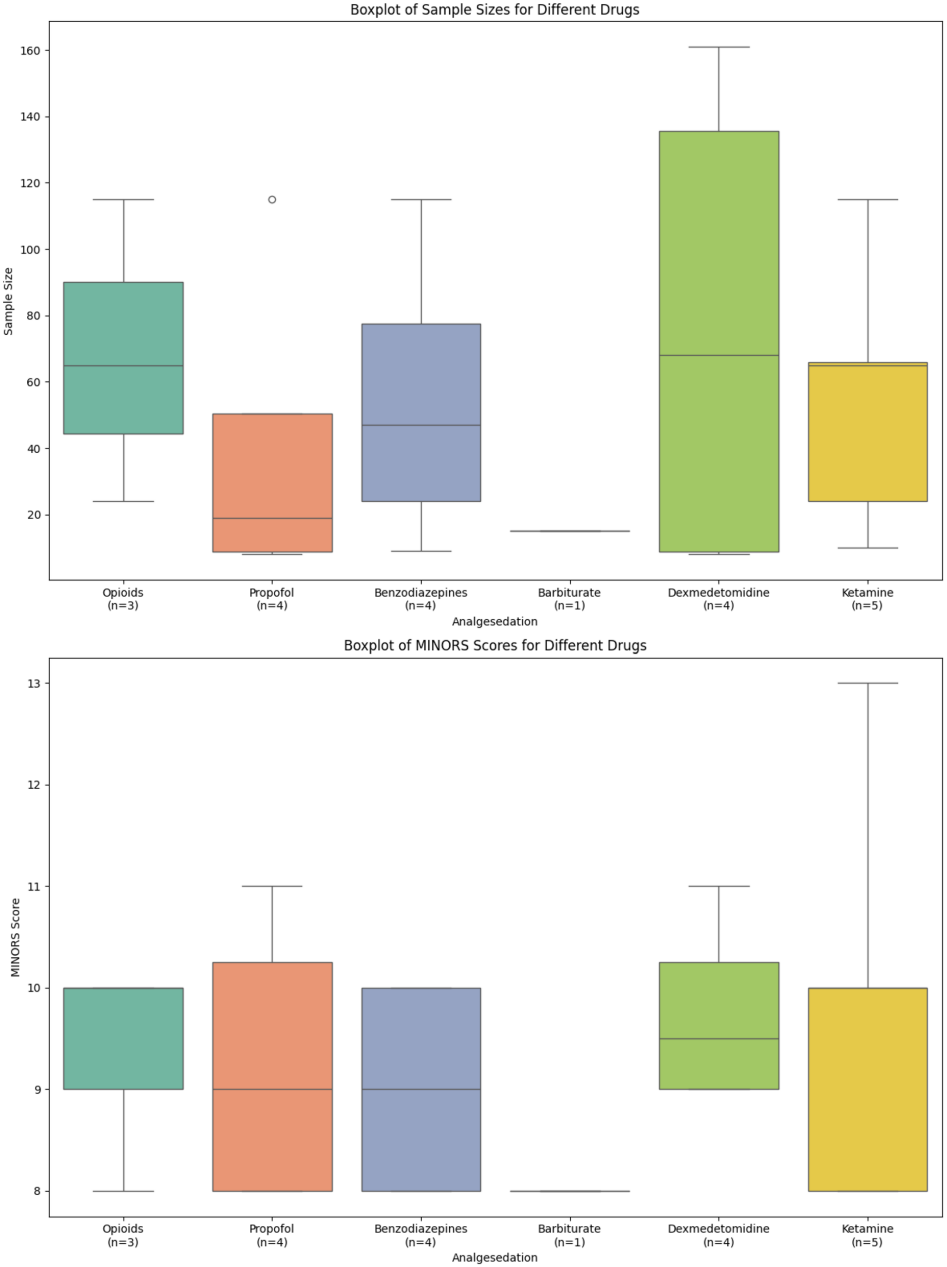
Boxplot of sample size and MINORS score of different drug class

Table 2 summarizes the study design, methodological characteristics, baseline population demographics, outcomes, and key findings observed in each study. The overall median sample size was 47 (interquartile range [IQR] 10–127) and median MINORS score was 9.5 (8–11). The studies’ publication years ranged from 1980 to 2023. There were six prospective studies [17–23] and five retrospective studies [20, 24–27]. Of these, there were four randomized studies [17–19, 22] and seven observational studies [20, 21, 23–27]. One study did not provide data on the age or sex of its participants. The median age of participants was 54 (50.15–58.1) years, and men constituted 46% of the study population (Fig. 4).

**Table 2:** Study design, methodological characteristics, baseline population characteristics, outcomes and key findings in each study

References	Sample size	Study Design	Outcomes and observations	Sedation Type	Sex	Mean age	MINORS score
Esfahani et al. [24]	127	Retrospective Observational Study	Vasospasm, GOS, mRS	dexmedetomidine	M 33, F 94	55.22	9
Okazaki et al. [25]	161	Retrospective Observational Study	DCI, vasospasm, ICU LOS, hospital LOS, mRS score	dexmedetomidine	M 51, F 110	62.3	9
Kallioinen et al. [17]	9	Prospective Randomised multiple cross-over trial	ICP, CPP	propofol, benzodiazepine, dexmedetomidine	M 5, F 4	58.1	10
James et al. [18]	8	Prospective Randomised control trial	ICP, CPP	dexmedetomidine, propofol	Not documented	Not documented	11
Schmittner et al. [19]	24	Prospective Randomised control trial	ICP, CPP, GOS	ketamine, opioid	M 15, F 9	50.15	10
Von der brelie et al. [20]	65	Retrospective Observational Study	DCI, 28-days mortality, ICU LOS, GOS, mRS	ketamine, opioid, benzodiazepine	M 29, F 36	58	10
Hertle et al. [21]	115	Prospective Observational Study	Spreading depolarisation	propofol, benzodiazepine, ketamine, opioid	M 69, F 46	49.6	8
Santos et al. [26]	66	Retrospective Observational Study	Spreading depolarisation	ketamine	M 24, F 42	53.45	8
Carlson et al. [22]	10	Prospective Randomised multiple cross-over trial	Spreading depolarisation	ketamine	M 3, F 7	60.8	13
Hertle et al. [27]	29	Retrospective Observational Study	GOS	propofol, benzodiazepine	M 12, F 17	54	8
Woodcock et al. [23]	15	Observational Study	ICP, CPP, 28-days mortality, GOS	barbiturate	M 11, F 4	42.8	8

### Narrative Summary

#### Dexmedetomidine

The use of the selective alpha 2 agonist, dexmedetomidine, was investigated in four of the studies that met inclusion criteria [17–20]. Basic physiological parameters were investigated by James et al. [18] in a small (*n* = 8) randomized unblinded crossover trial. No differences in ICP, CPP, heart rate, or microdialysate markers of tissue stress were found between dexmedetomidine (average dosage 0.54 ug/kg/hr) and propofol (average dosage 1.5 mg/hr). Cerebrovascular effects of dexmedetomidine were assessed by Kallioinen et al. [17] who measured static and dynamic cerebral autoregulation in the context of an increasing dosage regimen of dexmedetomidine (0.7–1 and then 1.4 ug/kg/hr). They found that at higher doses, dexmedetomidine was associated with dysfunction of dynamic cerebral autoregulation as measured by the transient hyperemic response ratio. Esfahani et al. [24] noted the potential benefits of dexmedetomidine which included amelioration of neuroinflammation and investigated the relationship between dexmedetomidine use and both vasospasm and neurological outcome. While the study collected data for 127 patients the single center retrospective design of the study, the variable dosing of dexmedetomidine and the lack of clear definitions of “vasospasm” make it difficult to assess the external validity of the study conclusion that dexmedetomidine use was associated with worse outcomes as measured by mRS and GOS. Okazaki et al. [25] performed a single center retrospective observational study examining the relationship between dexmedetomidine use and neurological outcome. On multivariable analysis designed to control for confounders they found that low dosage dexmedetomidine (0–0.2 ug/kg/hr) was associated with favorable neurological outcome at discharge (defined as mRS score of 0–2). This signal of benefit was not seen in the standard dosage (0.2–0.7 ug/kg/hr) dexmedetomidine group. The authors suggest that suppression of sympathetic activity (as indicated by a lower serum lactate level) may have been responsible for the improved outcomes, they also note a higher rate of adverse events such as hypotension in the higher dosage dexmedetomidine group. The signal for benefit in the low dosage group was relatively strong (odds ratio [OR] 3.17, 95% confidence interval [CI] 1.24–8.53, *p* = 0.02), but several methodological issues should be noted. This was a single center retrospective study with unblinded outcome assessment, further, the study only accounted for dexmedetomidine use in the initial 24 h of care. The result is also noteworthy because it identifies a clinical benefit from a dose range of dexmedetomidine that would result in relatively subtle clinical effects.

#### Ketamine

The use of ketamine in aSAH was investigated by five of the included studies. Schmittner et al. [19] investigated the use of S-ketamine in a mixed cohort of patients with acute brain injury (58% with aSAH). They compared the physiological effects of an analgosedative regime of S-ketamine and the barbiturate methohexitone with those of fentanyl and methohexitone and found no significant differences in ICP, CPP, or gut motility and a trend toward decreased vasopressor utilization in the ketamine group. These findings are reinforced by those of Von der Brelie et al. [20] who found that the use of ketamine sedation was associated with decreases in ICP, vasopressor use and the rate of DCI related cerebral infarction (7.3% in the ketamine sedation group vs. 25% in the nonketamine group). Hertle et al. [21] investigated the effect of ketamine and other sedatives on the incidence of CSD events, which are increasingly recognized as an important physiological mediator of DCI [28]. This retrospective cohort study of patients with acute brain injuries revealed a significant reduction in CSD incidence (OR 0.38, 95% CI 0.18–0.79, *p* = 0.01) and the number of CSD clusters (OR 0.2, 95% CI 0.06–0.64, *p* = 0.01). These findings are supported by those of Santos et al. [26] and Carlson et al. [22] who both found dose-dependent decreases in the incidence rate of CSDs with ketamine sedation regimens. The latter studies prospective randomized multiple crossover design provides strong evidence for a dosage range of 0.55–1.15 mg/kg/hr influencing CSD incidence with an OR of CSD occurrence of 13.838 (95% CI 1.99–1,000) for doses less than 1.15 mg/kg/hr. It is important to note that Carlson et al. [22] also found no increase in ICP associated with ketamine sedation.

#### Gamma-Aminobutyric Acid Agonist Sedatives: Propofol and Midazolam

The availability of evidence investigating propofol and midazolam in aSAH does not correspond to the frequency of their use for this pathology. Several of the identified studies investigated these medications, frequently as a comparator to the more novel sedatives outlined above. As noted above, James et al. [18] found no differences between propofol and dexmedetomidine infusions for heart rate, ICP, CPP, or cerebral microdialysate markers of tissue stress. Similarly, Kalioinen [17] found no disruption of static or dynamic cerebral autoregulation associated with the use of propofol or midazolam. Hertle et al. [27] performed a retrospective cohort study of the gamma-aminobutyric acid (GABA) sedatives propofol, midazolam and flunitrazepam and found a significant correlation between exposure to GABAergic sedatives (as measured by mean sedative scores) and poor neurological outcome as measured by GOS Extended. The authors note that no relationship was seen between sedative exposure and severity of initial disease (as measured by admission GCS) but caution that in-patient deterioration and complications represent a significant confounding factor that was not accounted for in their statistical analysis. Finally, Hertle et al. [21] found mixed evidence for the association of GABA agonist sedatives with incidence of CSD events. Propofol sedation was not associated with decreased CSD events but was found to be associated with a decrease in CSD clusters (OR 0.68, 95% CI 0.49–0.95, *p* = 0.03). Midazolam however was associated with an increased number of CSD events (OR 1.28, 95% CI 0.93–1.75, *p* = 0.13) and a significantly increased incidence of CSD clusters (OR 1.35, 95% CI 1–1.81, *p* = 0.048).

#### Opiates

Although opiates are commonly used in aSAH, there is limited evidence available demonstrating clinical benefit from this practice. As previously documented, Schmittner et al. [19] compared the use of fentanyl with that of ketamine and found no significant differences in ICP, CPP, or gut motility. Hertle et al. [21] investigated the use of fentanyl, sufentanil, and morphine and their association with CSD incidence and found no significant association. Overall, there were no signals of harm associated with the use of opiates in aSAH.

#### Barbiturates

There is minimal evidence for the use of barbiturates in aSAH. Woodcock et al. [23] report on the use of pentobarbitol bolus and infusion in the setting of raised ICP in a small (*n* = 15) mixed cohort of patients with acute brain injury (27% SAH). They document significant decreases in ICP for 5 of the 15 patients but validity is limited by the severity of neurological injury (all 15 patients had decerebrate or decorticate posturing, 12 patients exhibited 3rd cranial nerve palsies), the low proportion of patients with aSAH and the significant changes in clinical management practices in the 42 years since publication. Schmittner et al. [19] did include a barbiturate medication in their sedation protocols; however, this was not a variable of investigation, so it is not possible to draw meaningful conclusions regarding its use.

## Discussion

A systematic review of the literature was conducted to determine the effects of various analgosedative agents on patients with aSAH. A total of 11 articles met inclusion criteria with a median sample size of 29 (IQR 10–115) and median MINORS score of 9 (IQR 8–10). Two of the included studies were randomized controlled trials, two were randomized crossover trials and seven were retrospective observational studies.

This is the first study to collect and summarize the available evidence for the physiological effects of sedatives in aSAH. Key methodological points of note are the lack of high-quality evidence in the area and the variability in definitions of DCI. The available data indicate strong evidence for a dose-dependent reduction in the incidence of CSDs with the use of ketamine and does not demonstrate raised ICP in association with this medication. There is a potential signal of benefit from the use of low-dosage dexmedetomidine which may be mediated by attenuation of sympathetic activation and signals of harm associated with the use of midazolam in aSAH although the mechanism of this association is unclear. Propofol and opiates appear to be safe in the setting of aSAH but there is insufficient evidence to comment on the use of barbiturates.

The quality of evidence available for this central component of the management of aSAH in ICU is remarkably low. Methodological issues included retrospective and observational designs, small sample sizes, inadequate control of drug dosing, and insufficient control for confounding factors. Variability in the definitions used for DCI was also noted with outcome measures including angiographic and clinical vasospasm and DCI related cerebral infarction. Given the importance of DCI as a potentially modifiable mediator of outcome in aSAH we would emphasize the need for clear, evidence or consensus based [29] definitions of DCI (as exemplified by Von der Brelie’s definition of DCI related cerebral infarction [20]) to be used in future trials of analgosedatives in aSAH.

One significant issue with defining DCI as an outcome variable is the absence of a unifying mechanism linking the various features of the phenomenon, clinical deterioration, macrovascular, and microvascular vasospasm and radiographically confirmed cortical infarction. There is a growing evidence base that CSD events may be a pathophysiological mechanism linking these. CSDs co-occur with clinical deterioration of patients with aSAH [30] and direct measures of cortical ischemia [31]. They are also strongly associated with radiographically confirmed cortical infarction post aSAH [28]. This emerging picture of DCI underlines the importance of data demonstrating that ketamine sedation can ameliorate the incidence of CSD events in aSAH. These findings are supported by a convincing physiological mechanism whereby N-methyl-D-aspartate antagonists such as ketamine block glutamate driven depolarization events. This review has identified multiple studies demonstrating both dose-dependent reductions in CSD events and a reduction in DCI related cerebral infarction with the use of ketamine sedation. It should be noted that relatively high doses of ketamine are required to abolish CSD events completely and that the optimum level of CSD suppression is yet to be determined because CSD events may provide some neuroprotective benefit in healthy tissue [26]. Nevertheless, the availability of a drug with a favorable safety profile that may reduce DCI in aSAH and other forms of acute brain injury deserves further investigation and we anticipate the results of prospective interventional studies of ketamine in brain injury with great interest [32].

The use of dexmedetomidine and propofol in aSAH appears to be supported by available evidence in terms of safety and one study indicates that the use of low-dosage dexmedetomidine (0.01–0.2 µg/kg/hr) is associated with improved neurological outcomes [25]. Suggested mechanisms include reduced sympathetic activity and dampening of neuroinflammatory responses. Animal studies [33, 34] offer some evidence for these mechanisms, but robust supporting evidence from clinical trials is currently insufficient to draw conclusions about how these sedatives may ameliorate neurological injury. Although evidence of benefit requires further substantiation, this review reveals a favorable safety profile for these agents in aSAH. This is an important finding given that the rapid metabolism and short context sensitive half times of these drugs facilitate sedation breaks and clinical neurological assessment which are a vital screening tool for DCI. These factors strengthen arguments for the use of these sedatives in preference to benzodiazepines and longer acting opiates.

This review identified evidence of potential negative effects of benzodiazepines on patients with aSAH, associated with an increased incidence of DCI related cerebral infarctions [20] and spreading depolarization events [21]. One study also reported preliminary findings that GABAergic sedation led to significantly poorer outcomes in patients with aSAH [27]. Given the routine use of benzodiazepines in this patient group this finding deserves further exploration.

Neither opioids nor barbiturates have been shown to significantly affect outcomes in patients with aSAH regarding spreading depolarization [32], ICP, CPP, or GOS score. Given only three studies examined opioids [21–23], with only one of these being a randomized controlled trial [21], further research is required to draw definitive conclusions about these agents in the management of aSAH.

This review has a number of strengths; namely, we have used a robust and reproducible search strategy and followed the PRISMA guidelines. As such, we believe we have captured the entirety of the relevant aSAH specific literature. A number of limitations must also be acknowledged. The inclusion of retrospective and nonrandomized studies allows selection bias, and the lack of comparator control groups dilutes the strength of any conclusions. Inclusion of these studies also precludes quantitative synthesis of the data. This, however, represents the highly limited nature of the evidence currently available. The inclusion of a study from 1982 may also limit the validity of our findings, given the significant change in clinical management of aSAH since that time. It is important to note that only one study precedes 2007 [23]; after some discussion, the study was included to illustrate the limited evidence for barbiturate use in aSAH. Another limitation lies in the variability of definitions of DCI. When possible in our review, we have sought to document definitions used in the study for ease of interpretation. We also recognize the limitations of the other outcome measures used in the presented literature. Physiological parameters, such as ICP, CPP, and spreading depolarization, are clinically objective but may not impact long-term patient outcomes. Indeed, the lack of patient centeredness of physiological outcomes are common in the neurocritical care literature. Critically, this, and the paucity of data in general, underlines the necessity for well-designed, prospective clinical trials of sedatives in aSAH. Such studies should be adequately powered to determine how different sedative agents influence well defined patient-centered outcomes, such as 6-month GOS Extended score, thereby providing clearer guidance for the management of aSAH in clinical practice.

## Conclusions

This review highlights the very limited evidence that is available to inform the use of analgosedative medications in aSAH. This is a striking finding given the central role that these agents play in the management of more severe forms of the disease. Despite the limitations of this evidence, ketamine and dexmedetomidine emerge as agents that require further exploration, given their potential to reduce spreading depolarizations, macrovascular vasospasm, and DCI. The design and implementation of large-scale prospective studies with clearly defined patient-centered outcomes has the potential to significantly inform practice and improve outcomes from this devastating disease.

## Appendix

The Appendix of this research article, “The Impact of Sedative Choice in the Management of Aneurysmal Subarachnoid Hemorrhage: A Scoping Review,” includes detailed supplementary materials that support the main findings. It contains the search concepts and search strategy used to gather relevant studies, ensuring the reproducibility of the review process. Additionally, it features boxplots illustrating the sample sizes of different drugs and the Methodological Index for Nonrandomized Studies score associated with each drug. These visual representations provide a clearer understanding of the distribution and impact of various analgosedatives on patient outcomes.
